# ONeSAMP 3.0: estimation of effective population size via single nucleotide polymorphism data from one population

**DOI:** 10.1093/g3journal/jkae153

**Published:** 2024-07-12

**Authors:** Aaron Hong, Rebecca G Cheek, Suhashi Nihara De Silva, Kingshuk Mukherjee, Isha Yooseph, Marco Oliva, Mark Heim, Chris W. Funk, David Tallmon, Christina Boucher

**Affiliations:** Department of Computer and Information Science and Engineering, University of Florida, Gainesville, FL 32611, USA; Department of Biology, Colorado State University, Fort Collins, CO 80523, USA; Department of Computer and Information Science and Engineering, University of Florida, Gainesville, FL 32611, USA; Department of Computer and Information Science and Engineering, University of Florida, Gainesville, FL 32611, USA; Department of Computer and Information Science and Engineering, University of Florida, Gainesville, FL 32611, USA; Department of Computer and Information Science and Engineering, University of Florida, Gainesville, FL 32611, USA; Department of Math, Colorado State University, Fort Collins, CO 80523, USA; Department of Biology, Colorado State University, Fort Collins, CO 80523, USA; Biology and Marine Biology Program, University of Alaska Southeast, Juneau, AK 99801, USA; Department of Computer and Information Science and Engineering, University of Florida, Gainesville, FL 32611, USA

**Keywords:** effective population size, conservation, genetic diversity, single nucleotide polymorphism data

## Abstract

The genetic effective size (Ne) is arguably one of the most important characteristics of a population as it impacts the rate of loss of genetic diversity. Methods that estimate Ne are important in population and conservation genetic studies as they quantify the risk of a population being inbred or lacking genetic diversity. Yet there are very few methods that can estimate the Ne from data from a single population and without extensive information about the genetics of the population, such as a linkage map, or a reference genome of the species of interest. We present ONeSAMP 3.0, an algorithm for estimating Ne from single nucleotide polymorphism data collected from a single population sample using approximate Bayesian computation and local linear regression. We demonstrate the utility of this approach using simulated Wright–Fisher populations, and empirical data from five endangered Channel Island fox (*Urocyon littoralis*) populations to evaluate the performance of ONeSAMP 3.0 compared to a commonly used Ne estimator. Our results show that ONeSAMP 3.0 is broadly applicable to natural populations and is flexible enough that future versions could easily include summary statistics appropriate for a suite of biological and sampling conditions. ONeSAMP 3.0 is publicly available under the GNU General Public License at https://github.com/AaronHong1024/ONeSAMP_3.

## Teaser

The effective size (Ne) is crucial in population genetics for assessing genetic diversity loss. Estimating Ne are limited. Hong *et al*. introduce ONeSAMP 3.0, an algorithm using approximate bayesian computation and local linear regression to estimate Ne from single population SNP data without extensive genetic information. Tested on simulated and real data, ONeSAMP 3.0 outperforms traditional methods and is adaptable to various biological contexts. Moreover, with parallel processing, it is both time and memory efficient, allowing for estimations for large datasets.

## Introduction

Effective population size (Ne) is one of the most important parameters in population biology because it influences a host of evolutionary processes that affect population viability (Crow 1970; Lande *et al.* 1995; Lynch *et al.* 1995; Waples 2016a, 2016b). From a broad perspective, Ne can be defined as an “ideal” population that would experience drift at the same rate as the sample population (Waples 2022). As the number of potential parents in a population decreases, or variance of individual reproductive success increases, the Ne decreases (Wang *et al.* 2016). From an applied point of view, the Ne determines the rate of random change in allele frequencies and loss of genetic variation (Soule and Wilcox 1980; Frankham *et al.* 2014; Howard *et al.* 2017), and can be impacted by demographic and biological characteristics of a species (Ellegren and Galtier 2016; Hoban *et al.* 2020). Populations that have recently experienced a bottleneck event to a small Ne can have reduced fitness and elevated short-term extinction risk (Newman and Pilson 1997; Bijlsma *et al.* 2000). Furthermore, Ne may also inform whether a population can maintain adequate genetic variance for adaptive evolution in response to environmental change (Palstra 2008; Palstra and Fraser 2012). Estimating Ne is therefore of crucial importance in conservation and evolutionary biology.

The increasing availability of genetic data for many non-model organisms has catalyzed a tremendous effort in population genetics to accurately and precisely estimate Ne using molecular marker data (Wang *et al.* 2016). A number of useful genetic estimators have been developed to estimate Ne (e.g. Waples 2005; Nomura 2008; Zhdanova and Pudovkin 2008). A few approaches for estimating current Ne have been developed to take advantage of genomic datasets from a single sample of individuals (Wang 2009; Waples and Waples 2011; Barbato *et al.* 2015). One highly successful one-sample Ne estimator based on gametic disequilibrium is the software LDNe (Waples and Do 2008), included in the software NeEstimator (Do *et al.* 2014).

The use of summary statistics has been proposed as an alternative to metrics in population genetics that cannot efficiently be calculated exactly (Tavaré *et al.* 1997). These statistics offer a close approximation to their computationally intensive counterparts, and have proved to be successful in some empirical applications (Pritchard and Rosenberg 1999; Tishkoff *et al.* 2001; Nikolic and Chevalet 2014). Most applications have used a rejection sampling method (Pritchard and Rosenberg 1999), in which all summary statistic values that fall outside a given tolerance range are rejected, and only those summary statistics that fall within the tolerance range are used to estimate the target parameters (Tishkoff *et al.* 2001). In this paper, we present ONeSAMP 3.0, which uses local linear regression and smooth weighting to improve the reliability and accuracy of parameter estimation from summary statistics within an approximate Bayesian framework (Beaumont *et al.* 2002; Tallmon *et al.* 2008).

We combine multiple summary statistics in a Bayesian approach to capture the genotypic and allelic information contained in a single population genetic sample to estimate Ne (Beaumont *et al.* 2002; Tallmon *et al.* 2008). We simulate a Wright–Fisher (W–F) population model, incorporating a modification to account for distinct sexes, thereby ensuring that our estimate of effective population size (Ne) corresponds to a stable W–F population over a given number of generations, as determined by a prior distribution. Essentially, when our method deduces Ne from a sample, it aligns this estimation with the W–F Ne of the actual population from which the sample was derived, using the sample’s summary statistics for this determination. This approach means that, regardless of whether a population is growing, shrinking, or remaining stable, our method (ONeSAMP 3.0) accurately estimates the W–F equivalent Ne based on the gathered sample summary statistics. The original implementation of ONeSAMP 3.0 (Tallmon *et al.* 2008) used summary statistics and approximated Bayesian computation to estimate Ne from a single sample of microsatellite data. Here, we describe an updated and remodeled ONeSAMP 3.0 by adding new features which include the ability to accept single nucleotide polymorphism (SNP) data in GENEPOP format and data filtering algorithms to adjust for low coverage loci and individuals. We first outline the background of ONeSAMP 3.0’s approach in detail, before exploring the performance of ONeSAMP 3.0 compared to LDNe in replicate simulations with known Ne values and sample sizes typical of published SNP datasets. Finally, we apply both methods to data collected from Channel Island foxes to evaluate their performance on a dataset containing sub-populations that have experienced varying levels of population bottlenecks. ONeSAMP 3.0 gives a precise and reliable estimate of Ne for recently bottlenecked populations with appropriate sampling sizes. When the number of individuals sampled is less than 100, and the Ne value is relatively large (greater than or equal to 200), then ONeSAMP 3.0 is particularly useful. ONeSAMP 3.0 was implemented in python and went through rigorous usability testing. All source code and instructions for using ONeSAMP 3.0 are available on GitHub in anticipation that it will become an important part of the toolkit for researchers studying non-model organisms with limited sample sizes and numbers of loci genotyped.

## Materials and methods

### Background

ONeSAMP 3.0 estimates the Ne of an population using a similar paradigm as ONeSAMP (Tallmon *et al.* 2008). Briefly, it starts by simulating a large number of datasets with an identical number of individuals and loci as well as a known Ne value. Next, five statistics are computed for each of the simulated populations as well as the input population. Lastly, linear regression is used to infer the Ne value for the input population based on the statistics of the input population, and the known Ne value and statistics for the simulated populations. We note that ONeSAMP is restricted to microsatellite data and, thus, uses a subset of the same statistics as ONeSAMP 3.0. In contrast, ONeSAMP 3.0 has been redeveloped to use SNP data in GENEPOP format. In this section, we introduce these statistics used by ONeSAMP 3.0, and then describe the Bayesian model used to estimate Ne from the simulated populations.

### Calculation of summary statistics

Any summary statistics that can be calculated from standard population genetic data could be incorporated. We limited ONeSAMP 3.0 to a few that are straightforward to calculate, commonly used in population genetics studies, and related to Ne based upon previous results and simulations of our own. The five summary statistics incorporated in the ONeSAMP 3.0Ne estimator are calculated for the allelic or genotypic data from a sample of individuals genotyped at multiple loci. These summary statistics are defined as follows.

####  

##### Estimated mean expected heterozygosity

The first summary statistic calculated by ONeSAMP 3.0 is the estimated mean expected heterozygosity, denoted by ${\hat{H}}_{e}$. It has a positive relationship with effective population size and is the proportion of heterozygous genotypes expected under Hardy–Weinberg equilibrium (Nei 1987). ${\hat{H}}_{e}$ is also incorporated into many of the more complicated population genetics statistics used to infer population structure and dynamics so we begin with its definition below:


(1) $${\hat{H}}_{e}=\frac{1}{L}\sum_{i=1}^{L}{\hat{H}}_{e_{i}},$$

where *L* is the number of loci, and ${\hat{H}}_{e_{i}}$ is the heterozygous frequency of allele *i* in the population.

##### Fixation index

In a very small, non-selfing population, allele frequencies can differ between males and females, causing a heterozygote excess in their progeny over that expected based upon Hardy–Weinberg proportions. This quantity was estimated using the Wright–Fisher model calculated by dividing the observed heterozygosity by the expected heterozygosity. This is also referred to as the *F*-statistic or fixation index. It is denoted as ${\hat{F}}_{is}$ (Nei 1987) and is defined as follows:


(2) $${\hat{F}}_{is}=1-\frac{1}{L}\sum_{i=1}^{L}\frac{{\hat{H}}_{o_{i}}}{{\hat{H}}_{e_{i}}},$$

where the observed heterozygosities ${\hat{H}}_{o_{i}}$ and expected heterozygosities $H_{e_{i}}$ are averaged over all of the *L* loci.

##### Multi-locus homozygosity

Next, we used the first two moments of multi-locus homozygosity, the mean (denoted as $\hat{h}$) and variance (denoted as ${\hat{v}}_{h}$), as summary statistics. These can be calculated from counting the number of loci at which each of *S* individuals in a sample are homozygous (*h*).


(3) $$\hat{h}=\frac{1}{S}\sum_{\;j=1}^{S}h_{j},$$

(4) $${\hat{v}}_{h}=\frac{1}{S-1}\sum_{\;j=1}^{S}(h_{j}-\hat{h}{)}^{2}.$$

It has been shown that the moments of the distribution of an analogous measure, multi-locus heterozygosity, provide insights into Ne (Brown *et al.* 1980). Higher moments of multi-locus homozygosity were not used after initial simulations suggested that they were uninformative under the range of parameter values we examined.

##### Gametic disequilibrium

An estimate of gametic disequilibrium can be used to infer Ne when the reciprocal of the average of this statistic is taken over all pairs of distinct loci (Weir 1979). Non-random associations among alleles at different loci, or gametic disequilibria, are generated by finite Ne (Hill 1981). We calculate the square, ${\hat{r}}^{2}$, of Burrow’s estimator of gametic disequilibrium, $\hat{r}$, for the case where the phases of double heterozygotes are indistinguishable:


(5) $$\hat{r}=\frac{2}{L(L-1)}\sum_{1\leq i<j\leq L}\frac{\mathrm{freq}(AB)-A_{i}B_{j}}{\sqrt{\left(A_{i}(1-A_{i})+D_{i})(B_{j}(1-B_{j})+D_{j}\right)}},$$

where *L* is the number of loci, freq(AB) is the frequency of locus *A* and locus *B* occurring together, $A_{i}$ is the frequency of allele *A* at locus *i*, $B_{j}$ is the frequency of allele *B* at locus *j*, $D_{i}$ and $D_{j}$ are the respective departures of the loci from Hardy–Weinberg equilibrium, and i=1,…,L and j=1,…,L.

### Generating simulated populations

In ONeSAMP 3.0, we make use of an individual-based Wright–Fisher simulation model of a diploid species (Tallmon *et al.* 2004) with a target Ne. This model differs slightly from a Wright–Fisher model in that there are two allogamous sexes and equal numbers of each sex. This causes Ne to slightly exceed the sum of the number of males and females in the population (Balloux 2004). Given that we are trying to estimate current Ne, and the population may not be in equilibrium because of earlier demographic events, we allow for uncertainty in the initial conditions by initializing each iteration in the present model using a coalescent-based allele frequency distribution determined by a value randomly drawn from a uniform distribution. A population of size Ne is created and randomly mated for t∈[2,8] generations, again with *t* drawn from a uniform distribution. Progeny from the adults in generation *t* is sampled, and summary statistics are then calculated from this sample.

The simulation model samples each time from a uniform flat prior distribution for Ne∈[4,400] (except where specified as different; $\theta \in 4.8\times 10^{-5}$, $4.8\times 10^{-3})$, and duration in generations t∈[2,8] at size Ne, to generate $J=2\times 10^{4}$ values of the summary statistics. This prior is reasonable, as Ne can fall in this range, even for some populations with thousands of individuals, because Ne is often significantly less than the total population size (Palstra 2008).

### Estimating Ne using linear regression

In order to estimate the Ne value for the input population, we first simulate *J* populations as described in subsection and calculate the summary statistics for each population. We will denote the statistics for the *j*th population as $S_{j}$ for j=1,…,J. Hence, each $S_{j}$ is a vector of five real numbers. Next, we denote the summary statistics for the input population as $S^{*}$. We note that $S^{*}$ is also a vector of five real numbers. Next, we compute the Euclidean distance between the $S_{j}$ and $S^{*}$ for each j=1,…,J. All datasets that have an Euclidean distance less than *δ*, which is denoted as a distance threshold used to regulate the number of acceptable candidates, are used to estimate the target Ne value. This estimation is accomplished using weighted local regression. We refer the reader to Tallmon *et al.* (2008) for more details about this step. Finally, we apply a Box–Cox transformation (with λ=−0.2) to Ne across all regressions to guarantee that *θ*, as outlined in the preceding subsection, remains stable despite variations in *δ*. Values of Ne accepted within 0.02 are then regarded as samples from the posterior distribution of Ne.

### Implementation

ONeSAMP 3.0 is implemented in Python and R and is designed to be accessible to researchers with basic Linux or UNIX skills. All simulations were run on a server with AMD EPYC 7,702 CPU, running the Red Hat Enterprise Linux 7.7 (64-bit, kernel 3.10.0). Our method takes as input an input dataset in GENEPOP format, a lower Ne value (-lNe parameter), an upper Ne value (-uNe parameter), a minimum allele frequency (-m parameter), a value for the mutation rate (-r parameter), a lower and upper range for the population mutation rate (-lt and -ut parameters), lower and upper duration range (-ld and -ud parameters), the number of repeated simulation trials (-s parameter), the rate of missing data for individuals (-i parameter) and loci (-l parameter), and the input file name (-o parameter). We denote the population mutation rate as *θ* and note that it is a product of the mutation rate and the effective population size. The default values for these parameters are as follows: (1) the minimal allele frequency has a default value of 0.05, (2) the mutation rate has a default value of $1.2\times 10^{-8}$, (3) the default for the Ne range is [50,150], (4) the default range for the population mutation rate is $\left[4.8\times 10^{-3},4.8\times 10^{-5}\right]$, (5) the default number of trials is $2\times 10^{4}$, (6) the default duration range is [2,8], and lastly, (7) the default missing data rate is 20% for both individuals and loci. Using a combination of real and simulated SNP data, we compare the performance of ONeSAMP 3.0 to that of LDNe. Additionally, we implemented parallel processing to increase the time efficiency of ONeSAMP 3.0.

## Results

### Data description

We used various datasets to measure the performance of ONeSAMP 3.0 and LDNe. We first simulated data to determine and examine the reliability and accuracy of estimate for Ne based on the target Ne value, the number of loci, and the number of individuals. In particular, we simulated target populations with Ne equal to 100 or Ne equal to 200. For each target Ne, we explored the influence of sample size by generating datasets with samples from 50, 100, or 200 individuals and 40, 80, 160, or 320 loci. We used a uniform prior on Ne of [50, 150] or [20, 250] for the target Ne of 100. We used a uniform prior on Ne of [150, 250] for target Ne of 200. The variation in sample size and number of loci across different Ne values allows for the assessment to the impact of these factors on the reliability and accuracy of the estimates. This comprehensive approach is designed to provide insights into the most effective strategies for estimating Ne under various conditions.

### Accuracy of ONeSAMP 3.0 on data with a narrow Ne range

**Fig. 1.**
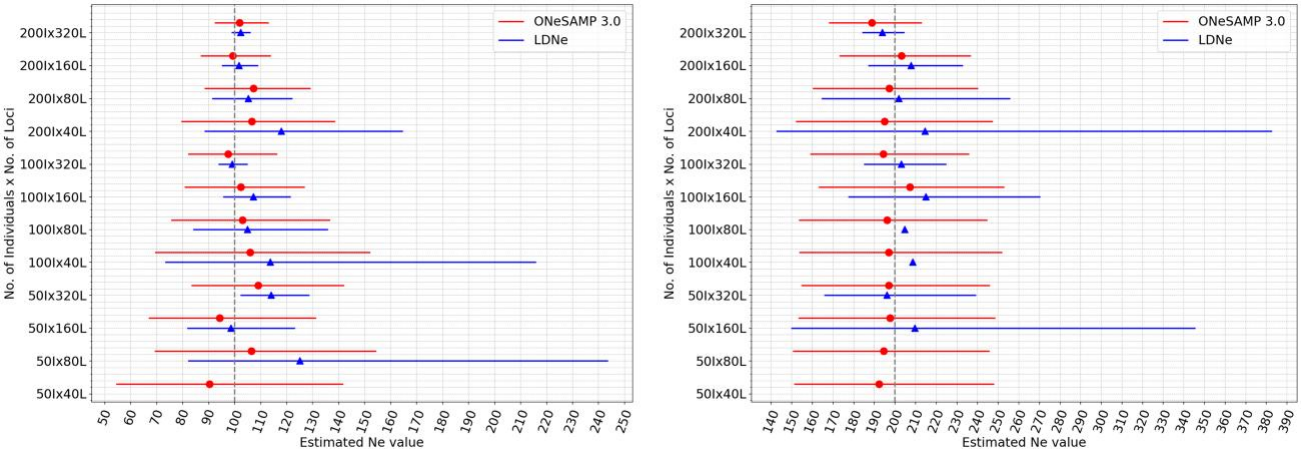
Comparison between the performance of ONeSAMP 3.0 and LDNe. ONeSAMP 3.0 is shown in circle, and LDNe is shown in triangle. The top figure shows results with the target Ne value 100. In this context, the lower limit (lNe) for Ne is 50, and the upper limit (uNe) is 150. The bottom figure shows results with the target Ne value 200. In this context, the lower limit (lNe) for Ne is 150, and the upper limit (uNe) is 250. Each point represents a predicted Ne value, and the horizontal line illustrates the confidence interval. Two adjacent horizontal lines indicate the Ne estimates for the same population. If a line is absent, it signifies an infinite confidence interval for that population.

Both ONeSAMP 3.0 and LDNe were able to infer the value of Ne for 12 datasets. To minimize bias, we simulated each set of simulated conditions 10 times, each time computing the median predicted Ne value along with the upper and lower 95% quantile. These results are shown in Fig. 1, which illustrates that ONeSAMP 3.0 consistently provided accurate predictions with narrow intervals for the upper and lower 95% quantile. We calculated the difference between the predicted Ne by ONeSAMP 3.0 and the target Ne value and the root mean squared error (RMSE) across all experiments with target Ne value of 100. When the number of individuals was 50, 100, and 200, the mean prediction of ONeSAMP 3.0 was 100.06, 102.26, and 103.78, with confidence intervals having sizes 73.98, 56.12, and 36.98, respectively. Hence, we witnessed that our method had increasingly narrow Ne confidence intervals as the number of individual increases.

Expanding our analysis to populations with a higher target Ne value of 200, Fig. 1 illustrates that as the number of individuals and loci increases, both ONeSAMP 3.0 and LDNe produce more precise predictions. ONeSAMP 3.0 performs well in scenarios with limited data. For example, with 50 individuals, with 100 individuals, and with 200 individuals and fewer than 320 loci, ONeSAMP 3.0 achieved results with an average difference of 2.29%, 0.585%, and 0.723% from the target Ne value, respectively. As previously seen with a target Ne value of 100, the confidence interval of ONeSAMP 3.0 decreases as the number of loci increases.

Next, we studied the size of the confidence interval with an increasing number of loci and a larger population size. The confidence interval of ONeSAMP 3.0 narrowed considerably with a larger number of loci. When the number of individuals was 200, we observed that the confidence interval had a steady decrease of 64.82% from 40 loci to 320 loci. Similarly, the confidence interval decreased from 82.85 to 34.26 when the number of loci went from 40 to 320 and the number of individuals was 100, and it decreased from 87.47 to 58.71 when the number of loci went from 40 to 320 and the number of individuals was 50. Thus, we see that the confidence intervals decreased when the number of individuals and/or the number of loci increased, which is reflective of the Law of Large Numbers (Dekking 2005), i.e. increasing the number of loci or individuals sampled will increase the convergence to the median Ne.

**Fig. 2.**
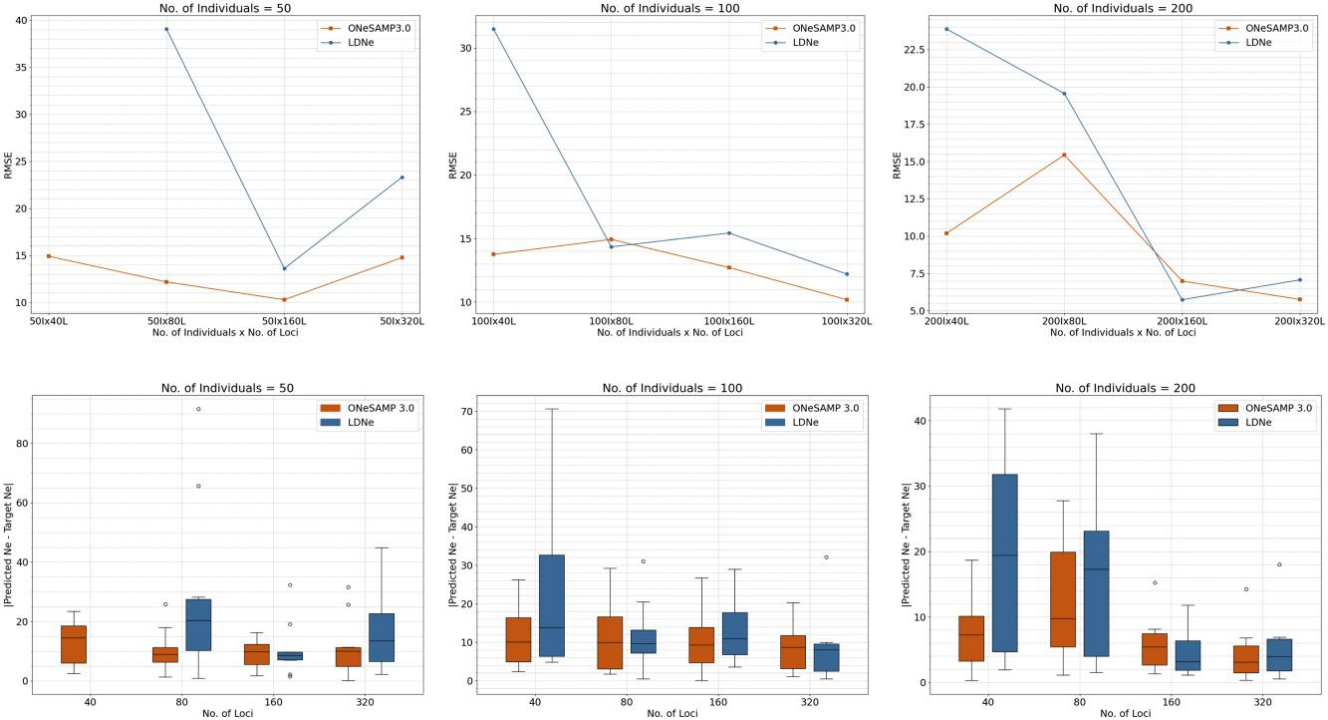
Illustration of the difference between the target Ne value and the predicted Ne value for varying numbers of loci and individuals. In the line graph, ONeSAMP 3.0 is shown in lower, and LDNe is shown in higher. In the box plot, ONeSAMP 3.0 is shown on the left, and the LDNe is shown on the right. The target Ne value is 100. The figures in the top row compare the RMSE values of the predictions made by ONeSAMP 3.0 and LDNe. The figures in the bottom row display the mean difference between 10 predictions from ONeSAMP 3.0 and LDNe. This mean difference is obtained by running both ONeSAMP 3.0 and LDNe 10 times on 12 different simulated datasets and then averaging the results.

Lastly, we calculated the RMSE between the predicted Ne value and the target Ne value, and determined how this parameter changes as the number of loci and individuals increases. We illustrate the trends in RMSE in Fig. 2, which displays RMSE value between the target Ne value and the predicted Ne value with increasing quantities of loci. We show that ONeSAMP 3.0 consistently produced 53.36%, 21.61%, and 18.78% smaller RMSE values than the LDNe. Furthermore, we observed that increasing the number of loci leads to improved reliability.

**Fig. 3.**
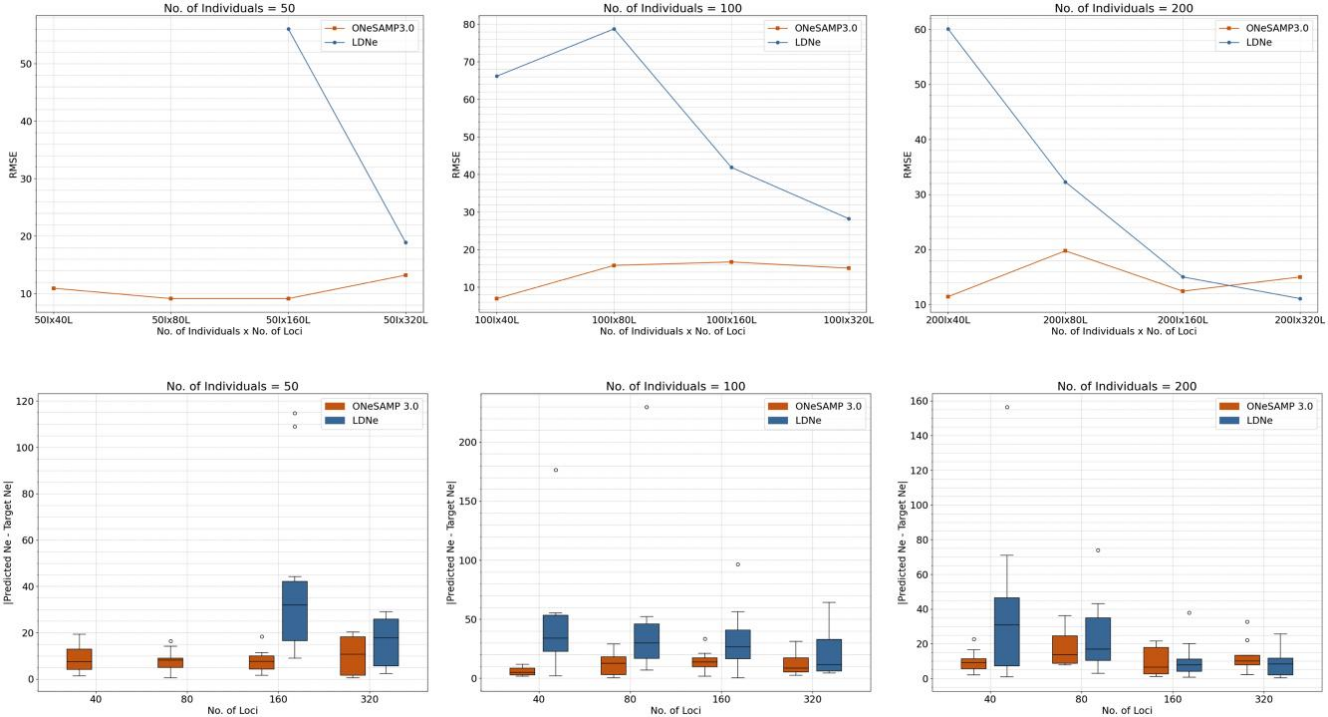
Illustration of the difference between the target Ne value and the predicted Ne value for varying numbers of loci and individuals. In the line graph, ONeSAMP 3.0 is shown in lower, and LDNe is shown in higher. In the box plot, ONeSAMP 3.0 is shown on the left, and the LDNe is shown on the right. The target Ne value is 200. The figures in the top row compare the RMSE values of the predictions made by ONeSAMP 3.0 and LDNe. The figures in the bottom row display the mean difference between 10 predictions from ONeSAMP 3.0 and LDNe. This mean difference is obtained by running both ONeSAMP 3.0 and LDNe 10 times on 12 different simulated datasets and then averaging the results.

Expanding our analysis to include target Ne value of 200, Fig. 3 shows that ONeSAMP 3.0 consistently produces 76.86%, 69.08%, and 25.39% smaller RMSE values than the LDNe, indicating its reliability in making predictions. Furthermore, this figure shows that ONeSAMP 3.0 yields consistently narrow confidence intervals and few outlier values. When the number of individuals was 200, we observed that the confidence interval had a steady decrease of 52.56% from 40 loci to 320 loci. Similarly, the confidence interval decreased from 98.21 to 77.04 when the number of loci went from 40 to 320 and the number of individuals was 100, and it decreased from 97.04 to 91.38 when the number of loci went from 40 to 320 and the number of individuals was 50.

### Accuracy of ONeSAMP 3.0 on data with a broad Ne range

**Fig. 4.**
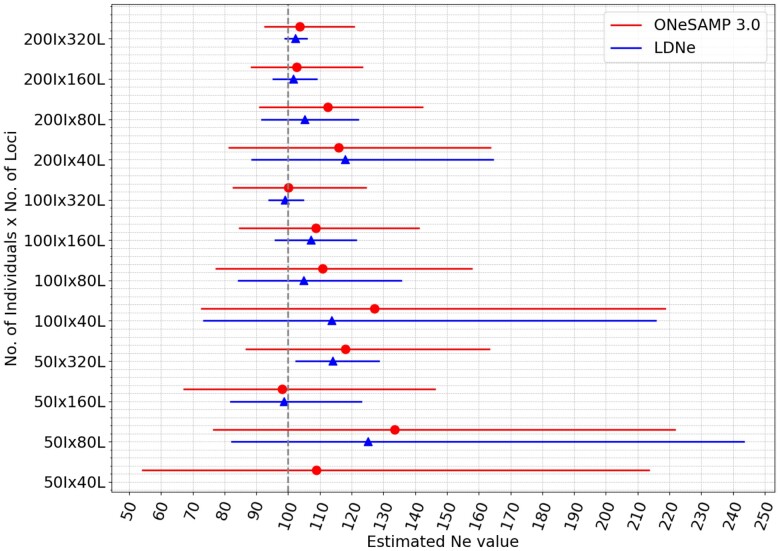
Estimator performance when Ne=100, Ne lower limit of 20, and Ne upper limit of 250. ONeSAMP 3.0 is shown in circle, and LDNe is shown in triangle. Each point corresponds to the median Ne value, and the horizontal line illustrates the confidence interval. Two adjacent horizontal lines depict the Ne estimates for the same population by ONeSAMP 3.0 (red) and LDNe (blue). The absence of a line signifies an infinite confidence interval for that population.

Based on the preceding experimental outcomes, we see that ONeSAMP 3.0 demonstrates consistent favorable performance across a variety of datasets. Motivated by these findings, we conducted an additional experimental inquiry aimed at elucidating the performance dynamics of ONeSAMP 3.0 under an extended scope of Ne values, spanning from 20 to 250. As illustrated in Fig. 4, it is discerned that in most cases, the prediction results of LDNe and ONeSAMP 3.0 are very close. Within the experimental context, when the number of individuals is 100 and the number of loci is 320, or when the individual size is 200 and the loci size is 40, ONeSAMP 3.0 outperforms LDNe. However, LDNe outperforms ONeSAMP 3.0 on all other datasets. More dramatic differences are shown between the confidence intervals with ONeSAMP 3.0 having considerably larger confidence intervals than LDNe which grow narrower as the number of individuals or loci increases. There was only a single dataset—with 50 individuals and 40 loci—where the LDNe cannot generate the confidence interval.

### Application of ONeSAMP 3.0 to Channel Island fox population data

To provide an example of the use and interpretation of ONeSAMP 3.0 as a genetic estimator of Ne, we reanalyzed data from six sub-populations of Channel Island foxes sampled across their range including San Miguel Island (SMI), Santa Rosa Island (SRI), Santa Cruz Island (SCI), San Nicolas Island (SNI), Santa Catalina Island (SCA), and San Clemente Island (SCL) at 4,860 SNPs (Funk *et al.* 2016). This dataset was chosen because several of the Channel Island fox sub-populations experienced population bottlenecks with varying levels of severity in the late 1990s due to heavy predation by invasive golden eagles (Roemer *et al.* 2001; Coonan *et al.* 2010) and disease (Timm *et al.* 2009). In this experiment, we first removed all loci containing missing data. The number of loci that remained after filtering is shown in Table 1. The SNI sub-population did not retain sufficient loci to be included in our analyses.

**Table 1.** Comparison between predictions reported by Funk *et al.* (2016) and ONeSAMP 3.0.

Population	*N*	No. loci	Ne from Funk *et al.* (2016)	Ne from LDNe	Ne from ONeSAMP3.0
SMI	24	943	13.70 (13.2–14.1)	6.8 (5.4–8.4)	8.5 (5.9–13.9)
SRI	23	1,692	13.6 (13.5–13.7)	11.2 (10.8–11.6)	17.0 (13.2–28.0)
SCI	24	1,778	25.1 (24.6–25.5)	21.2 (19.9–22.6)	23.7 (16.7–36.8)
SCA	46	605	47.0 (46.7–47.4)	44.3 (41.6–47.3)	66.8 (49.7–98.0)
SCL	19	805	89.7 (77.1–107.0)	30.9 (22.8–46.1)	76.1 (33.0–162.8)

For each population, the following is given: the number of individuals in each dataset (*N*), the number of loci after filtering (No. loci), the predicted effective population size reported by Funk *et al.* (2016), the effective population size predicted by LDNe, and the effective population size predicted by ONeSAMP 3.0. Alongside the predicted Ne values, confidence intervals are also provided. For these predictions, 300 loci was used.

**Fig. 5.**
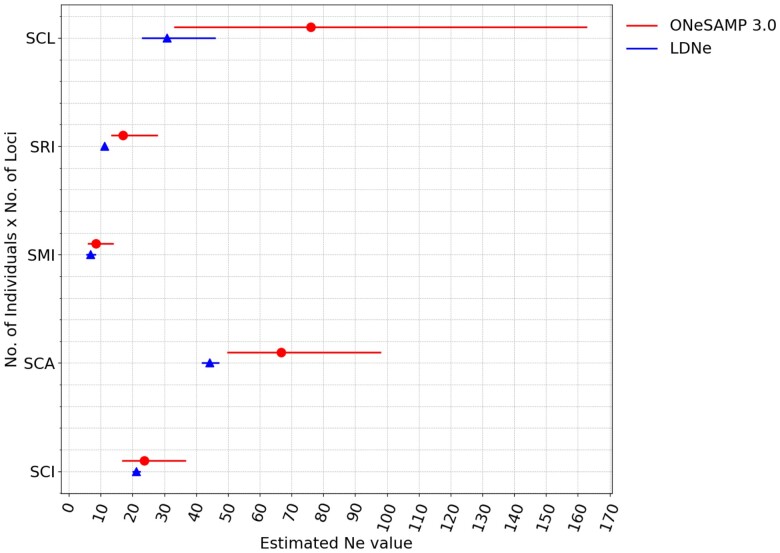
Results from ONeSAMP 3.0 and LDNe on the data collected from five distinct populations of island foxes with varying numbers of loci from each. For any given population, two adjacent horizontal lines compare Ne estimates obtained by ONeSAMP 3.0 and LDNe. ONeSAMP 3.0 is shown in circle, and LDNe is shown in triangle. Horizontal lines indicate the estimated range of Ne for each population, while pointers represent the median Ne estimate. It is emphasized that the analysis treats each of these populations as individual entities, deriving estimates from data specific to each.

Our results show that for all island datasets, ONeSAMP 3.0 produces estimates of Ne that are comparable to LDNe (see Fig. 5 and Table 1). To compare the prediction made by ONeSAMP 3.0 with LDNe, we calculated the difference between the predicted Ne value of ONeSAMP 3.0 with the Ne value provided by Funk *et al.* (2016), and similarly, the difference between the predicted Ne value of LDNe with the Ne value provided by Funk *et al.* (2016). We then considered the difference between the absolute value of these differences. Using this calculation, we determined that ONeSAMP 3.0 was closer to the Ne value reported by Funk *et al.* (2016) by 1.7, 2.5, and 45.2 for the SMI Island, SCI Island, and SCL Island datasets, respectively. Conversely, LDNe was closer to the Ne value reported by Funk *et al.* (2016) by 1.0 and 17.1 for the SRI Island and SCA Island datasets, respectively.

### Validation of prediction accuracy in simulated datasets

**Fig. 6.**
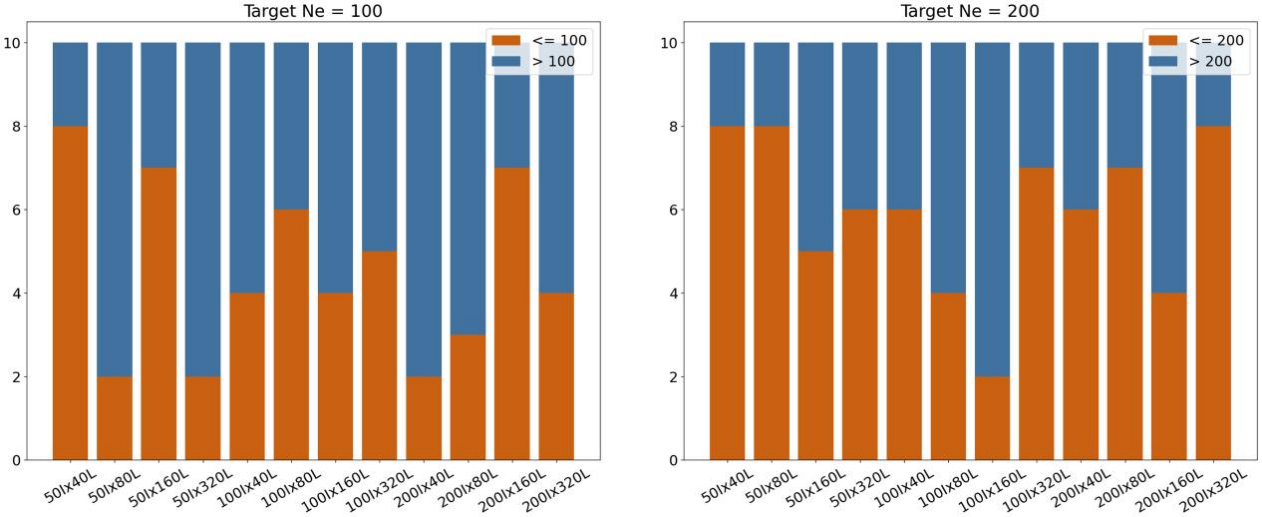
Illustration of the number of predicted Ne values that are higher or lower than the target Ne. The left figure shows experiments with a target Ne of 100, while the right figure shows experiments with a target Ne of 200.

In our simulated dataset experiments, we generated data 10 times for each combination of individuals and loci, and ran these datasets through ONeSAMP 3.0 to check for overestimates or underestimates in its predictions. The results indicated that for a target Ne of 100, ONeSAMP 3.0 produced 54 predictions below 100 and 64 predictions above 100. For a target Ne of 200, ONeSAMP 3.0 had 71 predictions below 200 and 49 predictions above 200. These findings suggest that ONeSAMP 3.0 does not have a significant bias toward overestimation or underestimation. The detailed results are shown in Fig. 6.

### Scalability of ONeSAMP 3.0

The scalability of the runtime of ONeSAMP 3.0 demonstrates a clear trend where the processing time increases with both the number of individuals and loci. For example, with 50 individuals, the runtime ranges from 23 s for 40 loci to 15 min and 29 s for 320 loci. This pattern continues as the number of individuals increases; for 100 individuals, the runtime starts at 26 s for 40 loci and extends to 16 min and 25 s for 320 loci. Similarly, for 200 individuals, the runtime ranges from 36 s for 40 loci to 25 min and 28 s for 320 loci. This progression indicates that the software requires more computational time as the dataset grows in size and complexity, maintaining a consistent scalability pattern across different dataset configurations. The memory usage was minimal; across all experiments, it was less than 110 MB. These results are illustrated in Table 2.

**Table 2.** Illustration of the computing resources needed for ONeSAMP 3.0 on increasingly larger datasets.

Dataset	Memory (MB)	Time (hh:mm:ss)
50 individuals × 40 loci	98.4	00:00:23
50 individuals × 80 loci	101.3	00:00:75
50 individuals × 160 loci	101.1	00:04:33
50 individuals × 320 loci	100.5	00:15:29
100 individuals × 40 loci	98.4	00:00:26
100 individuals × 80 loci	101.3	00:01:18
100 individuals × 160 loci	105.2	00:04:27
100 individuals × 320 loci	103.1	00:16:25
200 individuals × 40 loci	102.2	00:00:36
200 individuals × 80 loci	97.8	00:01:45
200 individuals × 160 loci	97.4	00:06:01
200 individuals × 320 loci	97.2	00:25:28

All experiments were ran with 64 threads.

## Discussion

We present ONeSAMP 3.0, which is a Bayesian computation method that predicts accurate estimates of Ne from a single population sample. Through simulations and empirical data, we demonstrate that ONeSAMP 3.0 is optimized to estimate Ne reliably even when there exists a limited number of loci and individuals. We demonstrated that the accuracy of ONeSAMP 3.0 has an average bias of 2.16% when the effective population size is 100, and an average bias of −1.63% when the effective population size is 200. The average bias is defined as 100 times the difference between the predicted effective population size and the target effective population size, divided by the target effective population size. Moreover, we showed that increasing the number of loci resulted in a consistent decrease in the width of the confidence interval. However, our experimental results indicate that in cases where the number of individuals is less than 100 and the number of loci is less than 160, the extended Ne range consistently leads to improved performance of the ONeSAMP 3.0 model. In optimal circumstances, the ONeSAMP 3.0 model demonstrates a significant enhancement, with augmentation rates between 20% and 83%.

Our results using empirical data from island fox sub-populations show that both ONeSAMP 3.0 and LDNe produced predictions of Ne that were close to those reported by Funk *et al.* (2016) for all island datasets. In the cases of SMI, SCI, and SCL islands, ONeSAMP 3.0 produced estimates for Ne that were closer to the previously published results for the island fox sub-populations compared to LDNe, which can be partially attributed to the generation of a large number of simulated populations, from which the probability distributions and related summary statistics of Ne under different scenarios are calculated. This Bayesian approach allows ONeSAMP 3.0 to integrate over the uncertainty in the parameters (i.e. target Ne values, values for *θ*, and mutation rate) to obtain a robust posterior distribution for Ne. This is in contrast to competing methods (such as LDNe), which only uses SNP data and/or linkage information (Do *et al.* 2014). Information on the size or history of a population can also be easily incorporated into ONeSAMP 3.0’s prior distribution for Ne by adding to the summary statistics calculating for each simulated population and input population. Moreover, because the prior distribution is generated by sampling simulated populations with the same number of loci and individuals as the empirical dataset, ONeSAMP 3.0 should mitigate the detrimental effects of small sample size on estimates of Ne. In addition, we note that one important feature of a Bayesian approach is that it limits the posterior confidence interval by use of a prior, which is required as input. The results reported here are directly from LDNe and ONeSAMP 3.0 when provided identical simulated and real datasets.

Next, we note that when new genotype or allele frequency summary statistics become available, it would be simple to adapt the approximate Bayesian method outlined here to estimate Ne. As an increasing number of molecular markers become available for a particular taxon, the approximate Bayesian framework provides an attractive and efficient method for estimating parameter values from the existing data. Nonetheless, it appears that reliable and exact Ne estimate is attainable using ONeSAMP 3.0 if moderate numbers of individuals and independent loci are sampled. Further research may also find more informative summary statistics to optimize this technique for application with various molecular marker types.

In conservation genetic practices, estimates of Ne provide researchers with important information for monitoring and recovery planning of populations (Luikart *et al.* 2010; Hoban *et al.* 2020). The general rule of thumb that an Ne of less than 50 should be considered a short-term extinction risk and an Ne above 500 poses no immediate genetic threat of extinction has garnered considerable support (Soule and Wilcox 1980; Jamieson and Allendorf 2012). However, a general problem for estimating Ne with genetic data is that each method has underlying assumptions regarding the sampled population’s demography and underlying population structure (Waples 2005; Wang *et al.* 2016). Therefore, each estimate of Ne must be interpreted with caution in cases of at-risk species regardless of the methodology. Lastly, we conclude by pointing out that it is important to correct Ne estimates for the number of chromosomes of the given study species when you estimate Ne for real populations. This can be accomplished using the corrections in Waples *et al.* (2016). Another option is to process the data so that the method only uses pairs of loci on different chromosomes.

### Recommendations

Our research findings suggest that the prediction of ONeSAMP 3.0 performs well when Ne value is large relative to the size of the dataset, i.e. as evaluated with Ne equal to 100 and Ne equal to 200. This becomes particularly pronounced when the dataset consists of a limited number of individuals (as evaluated with 50 individuals), a scenario where other methods may fail to produce reasonable estimates. On the other hand, the ONeSAMP 3.0 predictor can generate precise predictions in these instances. Our method relies on calculating linkage disequilibrium, in addition to expected heterozygosity, fixation index, and multi-locus homozygosity, for every simulated population and the sampled population. Since calculation of linkage disequilibrium is time consuming (it is more than 90% of the total CPU time), the running time of the method substantially increases as the number of loci increases. Whereas, LDNe only calculated linkage disequilibrium for the input population and, thus, is capable of handling a larger number of loci. For this reason, we recommend that LDNe be used when the dataset has a large number of loci. However, for studies where accuracy in estimating effective population size, especially in complex demographic scenarios, is paramount, our method’s comprehensive approach to calculating linkage disequilibrium across both input and simulated populations offers valuable insights. Future research could focus on optimizing these calculations to combine the thoroughness of our method with the efficiency of LDNe, bridging the gap between computational demand and analytical depth.

## Conclusion

We have demonstrated that ONeSAMP 3.0 can provide a more accurate prediction than LDNe for datasets containing less than 100 individuals and/or less than 100 loci. Hence, we anticipate that ONeSAMP 3.0 will become an important exploratory tool for estimating Ne in non-model species, or in scenarios when less than 100 individuals were capable of being sampled. The development of ONeSAMP 3.0 also opens the door for future considerations including the addition of statistics that mainly increase the accuracy of the prediction, and parallelization of the computation that will increase the efficiency of the tool. Additionally, the implementation allows for the use of other machine learning models in place of linear regression. Specifically, employing non-linear models—such as a random forest classifier or a convolutional neural network—could result in notable performance gains and increased precision.

## Data Availability

The datasets related to the fox are available at https://datadryad.org/stash/dataset/doi:10.5061/dryad.2kn1v. The authors confirm that all essential data required to validate the conclusions drawn in the article can be found within the article’s text, figures, and tables. Additionally, relevant code and supplementary materials can be accessed from our GitHub repository at https://github.com/AaronHong1024/ONeSAMP_3.
